# Analytic Function Approximation by Path-Norm-Regularized Deep Neural Networks

**DOI:** 10.3390/e24081136

**Published:** 2022-08-16

**Authors:** Aleksandr Beknazaryan

**Affiliations:** Institute of Environmental and Agricultural Biology (X-BIO), University of Tyumen, Volodarskogo 6, 625003 Tyumen, Russia; a.beknazaryan@utmn.ru

**Keywords:** deep neural networks, analytic functions, path norm regularization, exponential convergence

## Abstract

We show that neural networks with an absolute value activation function and with network path norm, network sizes and network weights having logarithmic dependence on 1/ε can ε-approximate functions that are analytic on certain regions of $C^{d}$.

## 1. Introduction

Deep neural networks have found broad applications in many areas and disciplines, such as computer vision, speech and audio recognition and natural language processing. Two of the main characteristics of a given class of neural networks are its complexity and approximating capability. Once the activation function is selected, a class of networks is determined by the specification of the network architecture (namely, its depth and width) and the choice of network weights. Hence, the estimation of the complexity of a given class is carried out by regularizing (one of) those parameters, and the approximation properties of obtained regularized classes of networks are then investigated.

The capability of shallow networks of depth 1 to approximate continuous functions is shown in the universal approximation theorem ([1]), and approximations of integrable functions by networks with fixed width are presented in [2]. Network-architecture-constrained approximations of analytic functions are given in [3], where it is shown that ReLU networks with depth depending logarithmically on 1/ε and width d+4 can ε-approximate analytic functions on the closed subcubes of ${\left(-1,1\right)}^{d}$.

The weight regularization of networks is usually carried out by imposing an $l_{p}$-related constraint on network weights, p≥0. The most popular types of such constraints include the $l_{0}$, $l_{1}$ and the *path norm* regularizations (see, respectively, [4,5,6] and references therein). Approximations of β-smooth functions on ${\left[0,1\right]}^{d}$ by $l_{0}$-regularized sparse ReLU networks are given in [5,7], and exponential rates of approximations of analytic functions by $l_{0}$-regularized networks are derived in [8].

Path-norm-regularized classes of deep ReLU networks are considered in [4], where, together with other characteristics, the Rademacher complexities of those classes are estimated. The network size independence of those estimates makes the path norm regularization particularly remarkable. As the estimation only uses the Lipschitz continuity (with Lipschitz constant 1), the idempotency and the non-negative homogeneity of the ReLU function, it can be extended to networks with the absolute value activation function. Network characteristics similar to the path norm are also considered in the works [9,10], where they are called, respectively, a *variation* and a *basis-path norm*, and statistical features of classes of networks are described in terms of those characteristics.

The objective of the present paper is the construction of path-norm-regularized networks that exponentially fast approximate analytic functions. Our goal is to achieve such convergence rates with activations that are idempotent, non-negative homogeneous and Lipschitz continuous with Lipschitz constant 1 so that the constructed path-norm-regularized networks fall within the scope of network classes studied in [4]. It turns out that networks with an absolute value activation function may suit this goal better than the networks with an ReLU activation function. More precisely, we show that analytic functions can be ε-approximated by networks with an absolute value activation function a(x) and with the path norm, the depth, the width and the weights all depending logarithmically on 1/ε. Such an approximation holds (i) on any subset ${\left(0,1-\delta \right]}^{d}\subset {\left(0,1\right)}^{d}$ for analytic functions on ${\left(0,1\right)}^{d}$ with absolutely convergent power series; (ii) on the whole hypercube ${\left[0,1\right]}^{d}$ for functions that can be analytically continued to certain subsets of $C^{d}$. Note that, as the network weights, as well as the total number of weights, depend logarithmically on 1/ε, then the $l_{1}$ weight norms of the constructed approximating deep networks are also of logarithmic dependence on 1/ε.

Note that the absolute value activation function considered in this paper is among the common built-in activation functions of the software-based neural network evolving method NEAT-Python ([11]). Training algorithms for networks with an absolute value activation function are developed in the works [12,13]. In addition, the VC-dimensions and the structures of the loss surfaces of neural networks with piecewise linear activation functions, including the absolute value function, are described in the works [14,15].

*Notation:* For a matrix $W\in R^{d_{1}\times d_{2}}$, we denote by $\left|W\right|\in R^{d_{1}\times d_{2}}$ the matrix obtained by taking the absolute values of the entries of *W*: ${\left|W\right|}_{ij}=\left|W_{ij}\right|$. For brevity of presentation, we will say that the matrix |W| is the *absolute value of the matrix W* (note that, in the literature, there are also other definitions of the notion of an absolute value of a matrix). The path norm of a neural network *f* is denoted by ${∥f∥}_{\times }$. For $x=\left(x_{1},\ldots ,x_{d}\right)\in R^{d}$ and $k=\left(k_{1},\ldots ,k_{d}\right)\in N_{0}^{d}$, the degree of the monomial $x^{k}=x_{1}^{k_{1}}\cdot \cdots \cdot x_{d}^{k_{d}}$ is defined to be ${∥k∥}_{1}=\sum_{i=1}^{d}k_{i}$. To assure that the matrix–vector multiplications are able to be accomplished, the vectors from $R^{d}$, according to the context, may be treated as matrices either from $R^{d\times 1}$ or from $R^{1\times d}$.

## 2. The Class of Approximant Networks

Neural networks are constituted of weight matrices, biases and nonlinear activation functions acting neuron-wise in the hidden layers. The biases, also called shift vectors, can be omitted by adding a fixed coordinate 1 to the input vector and correspondingly modifying the weight matrices. As the definition of the path norm of networks does not assume the presence of shift vectors, we will add a coordinate 1 to the input vector x and will consider classes of neural networks of the form

$$F_{\alpha }\left(L,p\right)=\left\{f:{\left[0,1\right]}^{p}\to R^{p_{L+1}}\;|\;\;f\left(x\right)=W_{L}\circ \alpha \circ W_{L-1}\circ \alpha \circ \cdots \circ \alpha \circ W_{0}\left(1,x\right)\right\},$$

where $W_{i}\in R^{p_{i+1}\times p_{i}}$ are the weight matrices, i=0,…,L, and $p=(p_{0},p_{1},\ldots ,p_{L+1})$ is the width vector, with $p_{0}=p+1$. The number of hidden layers *L* determines the depth of networks from $F_{\alpha }\left(L,p\right)$ and, in each layer, the activation function α:R→R acts element-wise on the input vector. For $f\in F_{\alpha }\left(L,p\right)$ given by

(1) $$f\left(x\right)=W_{L}\circ \alpha \circ W_{L-1}\circ \alpha \circ \cdots \circ \alpha \circ W_{0}\left(1,x\right),$$

let

(2) $${∥f∥}_{\times }:=∥\prod_{i=0}^{L}\left|W_{i}\right|{∥}_{1}$$

be the *path norm* of *f*, where ${∥\cdot ∥}_{1}$ denotes the $l_{1}$ norm of the $p_{0}\left(=p+1\right)$ dimensional vector $\prod_{i=0}^{L}\left|W_{i}\right|$ obtained as a product of absolute values of the weight matrices of *f*. For B>0, let

$$F_{\alpha }\left(L,p,B\right)=\{f\in F_{\alpha }{\left(L,p\right),∥f∥}_{\times }\leq B\}$$

be a path-norm-regularized subclass of $F_{\alpha }\left(L,p\right)$. As the results obtained in [4] indicate, the path norm regularizations are particularly well-suited for networks whose activation function α is

- Lipschitz continuous with Lipschitz constant 1;
- Idempotent, that is, α(α(x))=α(x), x∈R;
- Non-negative homogeneous, that is, α(cx)=cα(x), for c≥0, x∈R.

We therefore aim to choose an activation α possessing those properties such that analytic functions can be approximated by networks from $F_{\alpha }\left(L,p,B\right)$ with a small path norm constraint *B*. The most popular activation functions satisfying the above conditions are the ReLU function σ(x)=max{0,x} and the absolute value function a(x)=|x|. Below, we show that, with the absolute value activation function, the path norms of approximant networks may be significantly smaller than the path norms of the ReLU networks.

The standard technique of neural network function approximation relies on approximating the product function (x,y)↦xy, which then allows us to approximate monomials and polynomials of any desired degree. In [7], the approximation of the product $xy=({\left(x+y\right)}^{2}-x^{2}-y^{2})/2$ is achieved by approximating the function $x\mapsto x^{2}$. The latter is based on the observation that, for the triangle wave

(3) gs(x)=g∘g∘⋯∘g︸stimes1(x),

where g:[0,1]→[0,1] is defined by

$$g\left(x\right)=\left\{\begin{array}{cc} 2x, & 0\leq x<1/2,\\ 2(1-x), & 1/2\leq x\leq 1, \end{array}\right.$$

and for any positive integer *m*,

$$|x^{2}-f_{m}\left(x\right)|\leq 2^{-2m-2},$$

where

(4) $$f_{m}\left(x\right):=x-\sum_{s=1}^{m}\frac{g_{s}\left(x\right)}{2^{2s}}.$$

The approximation of $x^{2}$ by networks with the ReLU activation function σ(x) then follows from the representation

(5) g(x)=2σ(x)−4σ(x−1/2).

Thus, in this case, we will obtain matrices containing weights 2 and 4, which will make the path norm of approximant networks big. Note that the same approach is also used in [3] for constructing ReLU network approximations of analytic functions. In [5], the approximation of the product

$$\begin{array}{c} xy=h\left(\frac{x-y+1}{2}\right)-h\left(\frac{x+y}{2}\right)+\frac{x+y}{2}-\frac{1}{4} \end{array}$$

is achieved by approximating the function h(x):=x(1−x), which, in turn, is based on the observation that, for the triangle wave

$$\begin{array}{c} R^{k}=T^{k}\circ T^{k-1}\circ \cdots \circ T^{1}, \end{array}$$

where $T^{k}:\left[0,2^{2-2k}\right]\to \left[0,2^{-2k}\right]$ is defined by

(6) $$T^{k}\left(x\right):=\sigma \left(x/2\right)-\sigma \left(x-2^{1-2k}\right),$$

and for any positive integer *m*,

$$\begin{array}{c} |h\left(x\right)-\sum_{k=1}^{m}R^{k}\left(x\right)|\leq 2^{-m},\;x\in \left[0,1\right]. \end{array}$$

Although in the representation (6), the coefficients (weights) are all in [−1,1], the approximant $\sum_{k=1}^{m}R^{k}\left(x\right)$ in this case does not have the factors $2^{-2s}$ presented in the approximant $f_{m}\left(x\right)$ in (4), which, again, will result in big values of path norms. Therefore, in order to take advantage of the presence of those reducing weights, we would like to represent the function g(x) in (5) by a linear combination of activation functions with smaller coefficients. This is possible if, instead of σ(x), we deploy the absolute value activation function a(x). Indeed, in this case, we have that g(x) can be represented on [0,1] as

(7) g(x)=1−2a(x−1/2).

In the next section, we use the above representation (7) to show that analytic functions can be ε-approximated by networks from $F_{a}\left(L,p,B\right)$ with each of ${L,∥p∥}_{\infty }$ and *B*, as well as the network weights having logarithmic dependence on 1/ε. As all networks will have the same activation function a(x)=|x|, in the following, the subscript *a* will be omitted.

## 3. Results

We first construct a neural network with activation function a(x), that, for the given γ,m∈N, simultaneously approximates all *d*-dimensional monomials of a degree less than γ up to an error of $\gamma^{2}4^{-m}$. The depth of this network has order $m\log_{2}\gamma$ and its width is of order $m\gamma^{d+1}$. Moreover, the entries of the product of the absolute values of matrices of the network have an order of at most $\gamma^{5}$ (note the independence of *m*).

For γ>0, let $C_{d,\gamma }$ denote the number of *d*-dimensional monomials $x^{k}$ with degree ${∥k∥}_{1}<\gamma$. Then, $C_{d,\gamma }<{\left(\gamma +1\right)}^{d}$ and the following holds:

**Lemma** **1.** *There is a neural network* Mon*${}_{m,\gamma }^{d}\in F\left(L,p\right)$ with $L\leq \left\lceil \log_{2}\gamma \right\rceil \left(2m+5\right)+2$, $p_{0}=d+1$, $p_{L+1}=C_{d,\gamma }$ and ${∥p∥}_{\infty }\leq 6\gamma \left(m+2\right)C_{d,\gamma }$ such that*

$$∥{\mathrm{Mon}}_{m,\gamma }^{d}\left(x\right)-{\left(x^{k}\right)}_{{∥k∥}_{1}<\gamma }{∥}_{\infty }\leq \gamma^{2}4^{-m},\;x\in {\left[0,1\right]}^{d}.$$

*Moreover, the entries of the $C_{d,\gamma }\times \left(d+1\right)$-dimensional matrix obtained by multiplying the absolute values of matrices presented in ${\mathrm{Mon}}_{m,\gamma }^{d}$ are all bounded by $144{\left(\gamma +1\right)}^{5}$.*

Taking in the above lemma $\gamma ,m=\lceil \log_{2}\frac{1}{\varepsilon }\rceil$, we obtain a neural network from F(L,p), with *L* and ${∥p∥}_{\infty }$ having logarithmic dependence on 1/ε, which simultaneously approximates the monomials of a degree at most of γ with error ε (up to a logarithmic factor). Moreover, the entries of the product of absolute values of matrices of this network will also have logarithmic dependence on 1/ε. Below, we use this property to construct a neural network approximation of analytic and analytically continuable functions with an approximation error ε and with network parameters having logarithmic order.

**Theorem** **1.** *Let $f\left(x\right)=\sum_{k\in N_{0}^{d}}a_{k}x^{k}$ be an analytic function on ${\left(0,1\right)}^{d}$ with $\sum_{k\in N_{0}^{d}}\left|a_{k}\right|\leq F$. Then, for any ε,δ∈(0,1), there is a constant C=C(d,F) and a neural network $F_{\varepsilon }\in F\left(L,p,B\right)$ with $L\leq C\left(\log_{2}\frac{1}{\delta }\right)\left(\log_{2}^{2}\frac{1}{\varepsilon }\right),$${∥p∥}_{\infty }\leq \frac{C}{\delta^{d+1}}{\left(\log_{2}\frac{1}{\varepsilon }\right)}^{d+2}$ and*

$$B\leq 10^{4}dF{\left(\frac{\log_{2}\left(\left(2F+16\right)/\varepsilon \right)}{\delta }\right)}^{5},$$

*such that*

$$|F_{\varepsilon }\left(x\right)-f\left(x\right)|\leq \frac{\varepsilon }{\delta^{2}},\;\mathrm{forall}\;x\in {\left(0,1-\delta \right]}^{d}.$$

Note that an exponential convergence rate of deep ReLU network approximants on subintervals ${\left(0,1-\delta \right]}^{d}$ is also given in [3]. In our case, however, not only the depth and the width but also the path norm $∥F_{\varepsilon }{∥}_{\times }$ of the constructed network $F_{\varepsilon }$ have logarithmic dependence on 1/ε. Note that, in the above theorem, as δ approaches to 0, both ${∥p∥}_{\infty }$ and *B*, as well as the approximation error, grow polynomially on 1/δ. In the next theorem, we use the properties of Chebyshev series to derive an exponential convergence rate on the whole hypercube ${\left[0,1\right]}^{d}$. Recall that the Chebyshev polynomials are defined as $T_{0}\left(x\right)=1$, $T_{1}\left(x\right)=x$ and

$$T_{n+1}\left(x\right)=2xT_{n}\left(x\right)-T_{n-1}\left(x\right).$$

Chebyshev polynomials play an important role in the approximation theory ([16]), and, in particular, it is known ([17], Theorem 3.1) that if *f* is Lipschitz continuous on [−1,1], then it has a unique representation as an absolutely and uniformly convergent Chebyshev series

$$f\left(x\right)=\sum_{k=0}^{\infty }a_{k}T_{k}\left(x\right).$$

Moreover, in case *f* can be analytically continued to an ellipse $E_{\rho }\subset C$ with foci −1 and 1 and with the sum of semimajor and semiminor axes equal to ρ>1, then the partial sums of the above Chebyshev series converge to *f* with a geometric rate and the coefficients $a_{k}$ also decay with a geometric rate. This result was first derived by Bernstein in [18] and its extension to the multivariate case was given in [19]. Note that the condition $z\in E_{\rho }$ implies that $z^{2}\in N_{1,h^{2}}$, where $h=(\rho -\rho^{-1})/2$ and, for d,a>0, $N_{d,a}\subset C$ denotes an open ellipse with foci 0 and *d* and the leftmost point −a. For F>0, ρ>1 and $h=(\rho -\rho^{-1})/2$, let $A^{d}\left(\rho ,F\right)$ be the space of functions $f:{\left[0,1\right]}^{d}\to R$ that can be analytically continued to the region $\left\{z\in C^{d}:z_{1}^{2}+\cdots +z_{d}^{2}\in N_{d,h^{2}}\right\}$ and are bounded there by *F*. Using the extension of Bernstein’s theorem to the multivariate case, we obtain

**Lemma** **2.** *Let $\rho \geq 2^{\sqrt{d}}$. For $f\in A^{d}\left(\rho ,F\right)$, there is a constant C=C(d,ρ,F) and a polynomial*

$$p\left(x\right)=\sum_{{∥k∥}_{1}\leq \gamma }b_{k}x^{k},\;x\in {\left[0,1\right]}^{d},$$

*with*

(8) $$|b_{k}{|\leq C\left(\gamma +1\right)}^{d}$$

*and*

$$\left|f\left(x\right)-p\left(x\right)\right|\leq C\rho^{-\gamma /\sqrt{d}},\;\mathrm{forall}\;x\in {\left[0,1\right]}^{d}.$$

Combining Lemma 1 and Lemma 2, we obtain the following.

**Theorem** **2.** *Let ε∈(0,1) and let $\rho \geq 2^{\sqrt{d}}$. For $f\in A^{d}\left(\rho ,F\right)$, there is a constant C=C(d,ρ,F) and a neural network $F_{\varepsilon }\in F\left(L,p,B\right)$ with $L\leq C\log_{2}^{2}\frac{1}{\varepsilon },$${∥p∥}_{\infty }\leq C{\left(\log_{2}\frac{1}{\varepsilon }\right)}^{d+2}$ and $B\leq C{\left(\log_{2}\frac{1}{\varepsilon }\right)}^{2d+5}$ such that*

$$|F_{\varepsilon }{\left(x\right)-f\left(x\right)|\leq \varepsilon ,\;\mathrm{forall}\;x\in \left[0,1\right]}^{d}.$$

We conclude this part by estimating the $l_{1}$ weight regularization of networks constructed in Theorem 2. First, the total number of weights in those networks is bounded by ${\left(L+1\right)∥p∥}_{\infty }^{2}=O{\left(\log_{2}\frac{1}{\varepsilon }\right)}^{2d+6}.$ From (7), it follows that all of the weights of network ${\mathrm{Mon}}_{m,\gamma }^{d}$ from Lemma 1 are in [−2,2]. In Theorem 2, the network $F_{\varepsilon }$ is obtained by adding to a network ${\mathrm{Mon}}_{m,\gamma }^{d},$ with $\gamma =m=O(\log_{2}\frac{1}{\varepsilon })$, a layer with coefficients of partial sums of power series of an approximated function. Thus, using (8), we obtain that the $l_{1}$ weight norm of the network $F_{\varepsilon }$ constructed in Theorem 2 has order $O{\left(\log_{2}\frac{1}{\varepsilon }\right)}^{4d+6}$.

## 4. Proofs

In the following proofs, $I_{k}$ denotes an identity matrix of size k×k and all of the networks have activation a(x)=|x|. The proof of Lemma 1 is based on the following two lemmas.

**Lemma** **3.** *For any positive integer m, there exists a neural network* Mult*${}_{m}\in F\left(2m+3,p\right)$, with $p_{0}=3$, $p_{L+1}=1$ and ${∥p∥}_{\infty }=3m+2,$ such that*

(9) $$|{\mathrm{Mult}}_{m}\left(x,y\right)-xy|\leq 3\cdot 2^{-2m-3},\;\mathrm{for}\mathrm{all}\;x,y\in \left[0,1\right],$$

*and the product of absolute values of the matrices presented in* Mult*${}_{m}$ is equal to*

$$\left(3\sum_{k=1}^{m}\frac{2^{k}-1}{2^{2k}},2-2^{-m},2-2^{-m}\right).$$

**Proof.**  For k≥2, let $R_{k}$ denote a row of length *k* with a first entry equal to −1/2, last entry equal to 1 and all other entries equal to 0. Let $A_{k}$ be a matrix of size (k+1)×k obtained by adding the (k+1)-th row $R_{k}$ to the identity matrix $I_{k}$. That is,

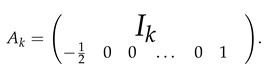

In addition, let $B_{k}$ denote a matrix of size k×k given by

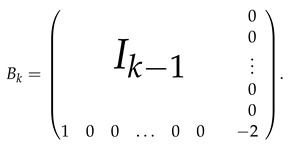

It then follows from (7) that

Bm+2∘a∘Am+1∘⋯∘B3∘a∘A21x=1xg1(x)g2(x)···gm(x),

where $g_{s}\left(x\right)$ is the function defined in (3), s=1,…,m. Thus, if $S_{m+2}$ is a row of length m+2 defined as

$$S_{m+2}=\left(0,1,-\frac{1}{2^{2\cdot 1}},-\frac{1}{2^{2\cdot 2}},\cdots ,-\frac{1}{2^{2\cdot m}}\right),$$

then

Sm+2∘a∘Bm+2∘a∘Am+1∘⋯∘a∘B3∘a∘A21x=fm(x),

where $f_{m}$ is defined by (4). We have that

$$|S_{m+2}\left|\cdot \right|B_{m+2}\left|\cdot \right|A_{m+1}\left|\cdot \cdots \cdot \right|B_{3}\left|\cdot \right|A_{2}|=\left(\sum_{k=1}^{m}\frac{2^{k+1}-2}{2^{2k}},2-2^{-m}\right).$$

As $xy=\frac{1}{2}\left({\left(x+y\right)}^{2}-x^{2}-y^{2}\right),$ then, in the first layer of ${\mathrm{Mult}}_{m}$, we will obtain a vector

$$\left(\begin{array}{ccc} 1 & 0 & 0\\ 0 & 1 & 0\\ 1 & 0 & 0\\ 0 & 0 & 1\\ 1 & 0 & 0\\ 0 & 1 & 1 \end{array}\right)\left(\begin{array}{c} 1\\ x\\ y \end{array}\right):=C\left(\begin{array}{c} 1\\ x\\ y \end{array}\right)=\left(\begin{array}{c} 1\\ x\\ 1\\ y\\ 1\\ x+y \end{array}\right)$$

and will then apply the network in a parallel manner from the first part of the proof to each of the pairs (1, *x*),(1, *y*) and (1, *x* + *y*). More precisely, for a given matrix *M* of size *p* × *q*, let $\tilde{M}$ be a matrix of size 3*p* × 3*q* defined as

$$\tilde{M}=\left(\begin{array}{ccc} M & 0 & 0\\ 0 & M & 0\\ 0 & 0 & M \end{array}\right).$$

Then, for the network

$${\mathrm{Mult}}_{m}\left(x,y\right)=\left(-\frac{1}{2},-\frac{1}{2},\frac{1}{2}\right)\circ a\circ {\tilde{S}}_{m+2}\circ a\circ {\tilde{B}}_{m+2}\circ a\circ {\tilde{A}}_{m+1}\circ \cdots \circ {\tilde{B}}_{3}\circ a\circ {\tilde{A}}_{2}\circ a\circ C\left(\begin{array}{c} 1\\ x\\ y \end{array}\right)$$

we have that

$${\mathrm{Mult}}_{m}\left(x,y\right)=\frac{1}{2}\left(f_{m}\left(x+y\right)-f_{m}\left(x\right)-f_{m}\left(y\right)\right),$$

which, together with $|f_{m}\left(x\right)-x^{2}|<2^{-2m-2}$ and the triangle inequality, implies (9). It remains to be noted that the product of absolute values of the matrices presented in ${\mathrm{Mult}}_{m}$ is equal to

$$\left(\frac{1}{2},\frac{1}{2},\frac{1}{2}\right)\cdot |{\tilde{S}}_{m+2}\left|\cdot \right|{\tilde{B}}_{m+2}\left|\cdot \right|{\tilde{A}}_{m+1}\left|\cdot \cdots \cdot \right|{\tilde{B}}_{3}\left|\cdot \right|{\tilde{A}}_{2}\left|\cdot |C\right|=\left(3\sum_{k=1}^{m}\frac{2^{k}-1}{2^{2k}},2-2^{-m},2-2^{-m}\right),$$

which completes the proof of the lemma. □

**Lemma** **4.** *For any positive integer m, there exists a neural network* Mult${}_{m}^{r}\in F\left(L,p\right)$*, with $L=\left(2m+5\right)\left\lceil \log_{2}r\right\rceil +1$, $p_{0}=r+1$, $p_{L+1}=1$ and ${∥p∥}_{\infty }\leq 6r\left(m+2\right)+1,$ such that*

$$|{\mathrm{Mult}}_{m}^{r}\left(x\right)-\prod_{i=1}^{r}x_{i}|\leq r^{2}4^{-m}\;for\;all\;\;\;x=\left(x_{1},\cdots ,x_{r}\right)\in {\left[0,1\right]}^{r},$$

*and, for the (r+1)-dimensional vector $J_{m}^{r}$ obtained by multiplication of absolute values of matrices presented in ${\mathrm{Mult}}_{m}^{r}$, we have that $∥J_{m}^{r}{∥}_{\infty }\leq 144r^{4}$.*

**Proof.**  First, for a given k∈N, we construct a network $N_{m}^{k}\in F\left(L,p\right)$ with L=2m+4,$p_{0}=2k+1$ and $p_{L+1}=k+1,$ such that

$$N_{m}^{k}\left(x_{1},x_{2},\ldots ,x_{2k-1},x_{2k}\right)=\left(1,{\mathrm{Mult}}_{m}\left(x_{1},x_{2}\right),\ldots ,{\mathrm{Mult}}_{m}\left(x_{2k-1},x_{2k}\right)\right).$$

In the first layer, we obtain a vector for which the first coordinate is 1 followed by triples $\left(1,x_{2l-1},x_{2l}\right)$l=1,…,k, that is, the vector $\left(1,1,x_{1},x_{2},1,x_{3},x_{4},\ldots ,1,x_{2k-1},x_{2k}\right)$. $N_{m}^{k}$ is then obtained by applying in parallel the network ${\mathrm{Mult}}_{m}$ to each triple $\left(1,x_{2l-1},x_{2l}\right)$ while keeping the first coordinate equal to 1. The product of absolute values of the matrices presented in this construction is a matrix of size (k+1)×(2k+1) having a form

$$\left(\begin{array}{cccccccccc} 1 & 0 & 0 & 0 & 0 & 0 & \ldots & 0 & 0 & 0\\ a_{m} & b_{m} & b_{m} & 0 & 0 & 0 & \ldots & 0 & 0 & 0\\ a_{m} & 0 & 0 & b_{m} & b_{m} & 0 & \ldots & 0 & 0 & 0\\ \cdot & \cdot & \cdot & \cdot & \cdot & \cdot & \cdot & \cdot & \cdot & \cdot \\ a_{m} & 0 & 0 & 0 & 0 & 0 & \ldots & 0 & b_{m} & b_{m} \end{array}\right),$$

where $a_{m}=3\sum_{k=1}^{m}\frac{2^{k}-1}{2^{2k}}$ and $b_{m}=2-2^{-m}$ are the coordinates obtained in the previous lemma. Let us now construct the network ${\mathrm{Mult}}_{m}^{r}$. The first hidden layer of ${\mathrm{Mult}}_{m}^{r}$ computes

(1,x1,…,xr)↦(1,x1,…,xr,1,1,…,1︸2q−r1),

where $q=\lceil \log_{2}r\rceil$. We then subsequently apply the networks $N_{m}^{2^{q}},N_{m}^{2^{q-1}},\ldots ,N_{m}^{2}$ and, in the last layer, we multiply the outcome by (0,1). From Lemma 3 and triangle inequality, we have that $|{\mathrm{Mult}}_{m}\left(x,y\right)-tz|\leq 3\cdot 2^{-2m-3}+|x-t|+|y-z|,$ for x,y,t,z∈[0,1]. Hence, by induction on *q*, we obtain that $|{\mathrm{Mult}}_{m}^{r}\left(x\right)-\prod_{i=1}^{r}x_{i}|\leq 3^{q}2^{-2m-3}\leq 3r^{2}2^{-2m-3}\leq r^{2}4^{-m}$.

Note that the product of absolute values of matrices in each network $N_{m}^{k}$ has the above form, that is, in each row, it has at most three nonzero values, each of which is less than 2. As the matrices given in the first and the last layer of ${\mathrm{Mult}}_{m}^{r}$ also satisfy this property, then each entry of the product of absolute values of all matrices of ${\mathrm{Mult}}_{m}^{r}$ will not exceed $12^{q+2}\leq 144r^{4}$. □

**Proof** **of** **Lemma** **1.** We have that, if ${∥k∥}_{1}=0$, then $x^{k}=1$, and if ${∥k∥}_{1}=1$, then k has only one non-zero coordinate, say, $k_{j}$, which is equal to 1 and $x^{k}=x_{j}$. Denote $N=C_{d,\gamma }-d-1$ and let $k^{1},\ldots ,k^{N}$ be the multi-indices satisfying $1<∥k^{i}{∥}_{1}<\gamma ,$i=1,…,N. For $k=(k_{1},\ldots ,k_{d})$ with ${∥k∥}_{1}>1$, denote by $x_{k}$ the $\left(∥k{∥}_{1}+1\right)$-dimensional vector of the form

xk=(1,x1,…,x1︸k11,…,xd,…,xd︸kd1).

The first layer of ${\mathrm{Mon}}_{m,\gamma }^{d}$ computes the $\left(d+1+\sum_{i=1}^{N}(∥k^{i}{∥}_{1}+1)\right)$-dimensional vector

$${\left(1,x,x_{k^{1}},\ldots ,x_{k^{N}}\right)}^{⊺}$$

by multiplying the input vector by matrix Γ of size $\left(d+1+\sum_{i=1}^{N}(∥k^{i}{∥}_{1}+1)\right)\times \left(r+1\right)$. In the following layers, we do not change the first d+1 coordinates (by multiplying them by $I_{d+1}$), and, to each $x_{k^{i}}$, we apply in parallel the network ${\mathrm{Mult}}_{m}^{∥k^{i}{∥}_{1}}$. Recall that, in Lemma 4, $J_{m}^{r}$ denotes the (r+1)-dimensional vector obtained from the product of absolute values of the matrices of ${\mathrm{Mult}}_{m}^{r}$. We then have that the product of the absolute values of the matrices of ${\mathrm{Mon}}_{m,\gamma }^{d}$ has the form

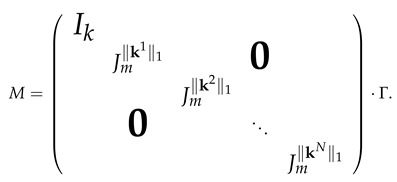

As the matrix Γ only contains entries 0 and 1, then, applying Lemma 4, we obtain that the entries of *M* are bounded by

$$\max_{1\leq i\leq N}||J_{m}^{∥k^{i}{∥}_{1}}|{|}_{1}\leq 144{\left(\gamma +1\right)}^{5}.$$

□

**Proof** **of** **Theorem** **1.** Let $\gamma =\left\lceil \frac{\log_{2}\left(\left(2F+16\right)/\varepsilon \right)}{\log_{2}{\left(1-\delta \right)}^{-1}}\right\rceil .$ Then, for $x\in {\left(0,1-\delta \right]}^{d}$, we have that

(10) $$|f\left(x\right)-\sum_{{∥k∥}_{1}<\gamma }a_{k}x^{k}|=|\sum_{{∥k∥}_{1}\geq \gamma }a_{k}x^{k}|\leq {\left(1-\delta \right)}^{\gamma }F\leq \frac{\varepsilon F}{2F+16}\leq \frac{\varepsilon }{2}\leq \frac{\varepsilon }{2\delta^{2}}.$$

Applying Lemma 1 with $m=\lceil \log_{2}\frac{4F+16}{\varepsilon }\rceil$, we obtain that, for all $x\in {\left[0,1\right]}^{d}$

(11) $$\begin{array}{cc} & ∥{\mathrm{Mon}}_{m,\gamma }^{d}\left(x\right)-{\left(x^{k}\right)}_{{∥k∥}_{1}<\gamma }{∥}_{\infty }\leq \gamma^{2}4^{-m}\leq \left(\frac{4}{\log_{2}^{2}{\left(1-\delta \right)}^{-1}}\right)\left(\log_{2}^{2}\frac{2F+16}{\varepsilon }\right){\left(\frac{\varepsilon }{4F+16}\right)}^{2}\\ \; & \leq \frac{4\left(2F+16\right)\varepsilon^{2}}{\delta^{2}\varepsilon {\left(4F+16\right)}^{2}}\leq \frac{\varepsilon }{2F\delta^{2}}, \end{array}$$

where we used the inequalities $\log_{2}{\left(1-\delta \right)}^{-1}\geq \delta ,\delta \in \left(0,1\right),$ and $\log_{2}^{2}r\leq r$ for r≥16. In order to approximate the partial sum $\sum_{{∥k∥}_{1}\leq \gamma }a_{k}x^{k},$ we add one last layer with the coefficients of that partial sum to the network ${\mathrm{Mon}}_{m,\gamma +1}^{d}$. As the sum of absolute values of those coefficients is bounded by *F*, then, combining (10) and (11), for the obtained network $F_{\varepsilon }$ we obtain

$$|F_{\varepsilon }\left(x\right)-f\left(x\right)|\leq \frac{\varepsilon }{\delta^{2}},\;for\;all\;x\in {\left(0,1-\delta \right]}^{d}.$$

From Lemma 1 it follows that

$$∥F_{\varepsilon }{∥}_{\times }\leq 144\left(d+1\right)F{\left(\gamma +1\right)}^{5}\leq 10^{4}dF{\left(\frac{\log_{2}\left(\left(2F+16\right)/\varepsilon \right)}{\delta }\right)}^{5}.$$

□

Let us now present the result from [19] that will be used to derive Lemma 2. First, if $f\in A^{d}\left(\rho ,F\right)$, then ([20], Theorem 4.1) *f* has a unique representation as an absolutely and uniformly convergent multivariate Chebyshev series

$$f\left(x\right)=\sum_{k_{1}=0}^{\infty }\ldots \sum_{k_{d}=0}^{\infty }a_{k_{1},\ldots ,k_{d}}T_{k_{1}}\left(x_{1}\right)\ldots T_{k_{d}}\left(x_{d}\right),\;x\in {\left[0,1\right]}^{d}.$$

Note that, for $k:=(k_{1},\ldots ,k_{d})$, the degree of a *d*-dimensional polynomial $T_{k_{1}}\left(x_{1}\right)\ldots T_{k_{d}}\left(x_{d}\right)$ is ${∥k∥}_{1}=k_{1}+\cdots +k_{d}$. Then, for any non-negative integers $n_{1},\ldots ,n_{d},$ the partial sum

(12) $$p\left(x\right)=\sum_{k_{1}=0}^{n_{1}}\ldots \sum_{k_{d}=0}^{n_{d}}a_{k}T_{k_{1}}\left(x_{1}\right)\ldots T_{k_{d}}\left(x_{d}\right)$$

is a polynomial truncation of the multivariate Chebyshev series of *f* of degree $d\left(p\right)=n_{1}+\cdots +n_{d}$. It is shown in [19] that

**Theorem** **3.** *For $f\in A^{d}\left(\rho ,F\right)$, there is a constant C=C(d,ρ,F) such that the multivariate Chebyshev coefficients of f satisfy*

(13) $$|a_{k}|\leq C\rho^{-{∥k∥}_{2}}$$

*and, for the polynomial truncations p of the multivariate Chebyshev series of f, we have that*

$$\underset{d(p)\leq \gamma }{\inf}{∥f\left(x\right)-p\left(x\right)∥}_{{\left[0,1\right]}^{d}}\leq C\rho^{-\gamma /\sqrt{d}}.$$

**Proof** **of** **Lemma** **2.** Note that, from the recursive definition of the Chebyshev polynomials, it follows that, for any k≥0, the coefficients of the Chebyshev polynomial $T_{k}\left(x\right)$ are all bounded by $2^{k}$. Let *p* now be a polynomial given by (12) with degree d(p)≤γ. As the number of summands in the right-hand side of (12) is bounded by ${\left(\gamma +1\right)}^{d}$, then, using (13), we obtain that *p* can be rewritten as

$$p\left(x\right)=\sum_{{∥k∥}_{1}\leq \gamma }b_{k}x^{k},$$

with

$$|b_{k}{|\leq C\left(\gamma +1\right)}^{d}2^{{∥k∥}_{1}}\rho^{-{∥k∥}_{2}}\leq C{\left(\gamma +1\right)}^{d}2^{\sqrt{d}{∥k∥}_{2}}\rho^{-{∥k∥}_{2}}\leq C{\left(\gamma +1\right)}^{d},$$

where the last inequality follows from the condition $\rho \geq 2^{\sqrt{d}}$. □

**Proof** **of** **Theorem** **2.** The proof follows from Lemmas 1 and 2 by taking $\gamma =m=\lceil \log_{2}\frac{1}{\varepsilon }\rceil$ and adding, to the network ${\mathrm{Mon}}_{m,\gamma +1}^{d}$, the last layer with the coefficients of the polynomial p(x) from Lemma 2. For the obtained network $F_{\varepsilon }$ we have that

$$∥F_{\varepsilon }{∥}_{\times }\leq 144C\left(d+1\right)C_{d,\gamma +1}{\left(\gamma +2\right)}^{d}{\left(\gamma +2\right)}^{5}\leq 144C\left(d+1\right){\left(\gamma +2\right)}^{2d+5},$$

where *C* is the constant from Lemma 2. □

## 5. Discussion

Although various activation functions, including the ReLU, sigmoid and the Gaussian function, have already been used in the literature for neural network approximations of smooth and analytic functions (see [3,8,21]), approximating properties of neural networks with an absolute value activation function, which is a built-in activation function of software-based neural network evolving methods (such as NEAT-Python, [11]), has been barely covered previously. Whereas the algorithms developed in the works [12,13] allow us to train neural networks with an absolute value activation function, in the present paper, we study the capabilities of those networks to approximate analytic functions. While popular types of constraints imposed on approximating neural networks are either controlling the $l_{p}$ norms of network weights or adjusting their architectures, in the present work, we study approximating properties of neural networks with regularized path norms and show that networks with an absolute value activation function and with network path norms having logarithmic dependence on 1/ε can ε-approximate functions that are analytic on certain regions of $C^{d}$. The sizes and the weights of constructed networks also have logarithmic dependence on 1/ε.

## Data Availability

Not applicable.
